# Differential Associations Between Volumes of Atrophic Cortical Brain Regions and Memory Performances in Early and Late Mild Cognitive Impairment

**DOI:** 10.3389/fnagi.2019.00245

**Published:** 2019-09-04

**Authors:** Dong Woo Kang, Hyun Kook Lim, Soo-hyun Joo, Na Rae Lee, Chang Uk Lee

**Affiliations:** ^1^Department of Psychiatry, Seoul St. Mary’s Hospital, College of Medicine, The Catholic University of Korea, Seoul, South Korea; ^2^Department of Psychiatry, Yeouido St. Mary’s Hospital, College of Medicine, The Catholic University of Korea, Seoul, South Korea

**Keywords:** voxel-based morphometry, early mild cognitive impairment, late mild cognitive impairment, memory performance, gray matter volume

## Abstract

**Background:**

Early and late mild cognitive impairment (MCI) patients have been reported to have a distinctive prognosis of converting to Alzheimer’s disease.

**Objective:**

To evaluate the difference in gray matter volume and assess the association between cognitive function evaluated by comprehensive cognitive function test, and cortical thickness across healthy controls (HCs) (*n* = 37), early (*n* = 30), and late MCI patients (*n* = 35).

**Methods:**

Differences in gray matter volume were evaluated by whole brain voxel-based morphometry across the groups. Multiple regression analysis was used to analyze group by memory performance interactions for the normalized gray matter volume.

**Results:**

The early MCI group showed reduced gray matter volume in the right middle temporal gyrus in comparison to the HC group. The late MCI group displayed atrophy in the left parahippocampal gyrus in comparison to the HC group. Late MCI patients exhibited a decreased gray matter volume in the left fusiform gyrus in comparison to patients in the early MCI group (Monte Carlo simulation corrected *p* < 0.01, Tukey *post hoc* tests). Furthermore, there was a significant group (HC vs. early MCI) by memory performance interaction for the normalized cortical volume of the right middle temporal gyrus. Additionally, a significant group (early MCI vs. late MCI) by memory performance interaction was found for the normalized gray matter volume of the left fusiform gyrus (*p* < 0.001).

**Conclusion:**

Early and late MCI patients showed distinctive associations of gray matter volumes in compensatory brain regions with memory performances. The findings can contribute to a better understanding of the structural changes in compensatory brain regions to elucidate memory decline in the trajectory of the subdivided prodromal stages of the Alzheimer’s disease (AD).

## Introduction

Mild cognitive impairment (MCI) is a feature of the transitional stage from normal aging to Alzheimer’s disease (AD) (Petersen et al., 2001), which has been reported to progress to dementia at a rate of 10–15% per year (Gauthier et al., 2006). Moreover, when memory is deteriorated in consideration of age, sex, and years of education, it is classified as an amnestic subtype of MCI (Petersen and Negash, 2008), which has been shown to be associated with beta-amyloid (Aβ) retention (Markesbery et al., 2006). Amnestic MCI is classified into early and late MCI based on the degree of deterioration in memory performances evaluated by detailed neuropsychological tests (Jessen et al., 2014). In addition, the early MCI has been suggested to reflect individuals at an earlier point in the clinical spectrum of AD compared with the late MCI (Aisen et al., 2010). Furthermore, recent studies have demonstrated that the risk of AD increases in patients with late MCI in comparison to patients with early MCI (Jessen et al., 2014).

In the trajectory of AD, cortical brain atrophy is preceded by Aβ accumulation and tauopathy (Jack et al., 2013). Moreover, atrophy has been described to progress at different rates depending on the region of the brain (Schuff et al., 2012) and to follow a sigmoidal shape as a function of time (Sabuncu et al., 2011). Given this AD pathogenesis, cortical changes have been documented to have an association with functional changes in brain and cognitive changes (Buckner et al., 2005).

Previous research has reported the structural change in the brain at the prodromal stage of the AD. Amnestic MCI subjects have shown the atrophy in hippocampal region, cingulate gyri, temporoparietal, and frontal neocortex in comparison to cognitively normal elderly and have displayed the preserved cortical volume in posterior association cortex in comparison to AD patients (Chetelat et al., 2002; Pennanen et al., 2005; Hämäläinen et al., 2007; Yao et al., 2011). In one study evaluating cortical changes in early and late MCI, a late MCI group showed atrophy in the entorhinal cortex, parahippocampal gyrus, and inferior temporal gyrus in comparison to an early MCI group. The early MCI group exhibited increased volume in the precuneus, supramarginal, and superior parietal gyrus in comparison to healthy elderly subjects and the late MCI group (Li et al., 2017). These structural changes have been documented to be related to memory, attention, and executive function in several regions of interest including the middle frontal gyrus, parahippocampal gyrus, and fusiform gyrus (Bonekamp et al., 2010). However, in this previous study, the results should be interpreted considering that the mean age of early MCI patients was profoundly lower than late MCI patients and controls. In contrary to this previous result, another previous study has reported the temporal atrophy, which is modulated by *APOE* ε4 genotype, in early MCI patients compared with healthy controls (HCs) (Risacher et al., 2013).

In addition, the other previous study has differentiated MCI converters from non-converters by evaluating hemispheric asymmetry of morphological biomarker including the cortical thickness in the medial temporal lobe (Long et al., 2018). Although the differentiation by MCI converter and non-converter is more clinically meaningful than that by early and late MCI, the former differs from the latter that can be examined at specific time points in that long-term follow-up observations are required for evaluating MCI converter and non-converter.

In these previous studies, however, atrophy of the cortex has been assessed only for specific brain regions of interest, without evaluation across the whole brain in early and late MCI. In addition, although the deterioration of cognitive function in various domains was observed in the MCI stage (Mistridis et al., 2015), most of the previous studies have mainly focused on a domain of memory. Additionally, although early MCI and late MCI reflect different stages in the clinical spectrum of AD, few studies have examined a differential relationship between cortical atrophy and cognitive decline in early and late MCI.

The aim of this study is to evaluate the difference in gray matter volume by whole brain voxel-based morphometry and to assess the association between cognitive function evaluated by comprehensive cognitive function test, and cortical thickness across cognitively normal older adults, early, and late MCI patients. As previous studies have reported the different prognosis and the difference in cortical atrophy, and cognitive decline in the subdivided prodromal stages of the AD, we hypothesized that there would be a significant difference in cortical thickness between these diagnoses in the medial temporal lobe and that there would be a distinctive association between cortical atrophy and cognitive decline among controls, early, and late MCI.

## Materials and Methods

### Subjects

One hundred two subjects were included in this study [37 subjects in the healthy control (HC) group (age range: 72–78 years), 30 subjects with early MCI (age range: 71–82 years), and 35 subjects with late MCI (age range: 69–82 years)]. Subjects were recruited from the Catholic Geriatric Brain MRI database, which was built through the outpatient psycho-geriatric clinic of Seoul Saint Mary’s Hospital located in Seoul, Republic of Korea, from October 2016 to July 2018. The cognitive functions of all subjects were assessed with the Korean version of the Consortium to Establish a Registry for AD (CERAD-K) (Lee et al., 2002). Measures included assessment in verbal fluency (VF), the 15-item Boston Naming Test (BNT), the Korean version of the Mini-Mental State Examination (MMSE-K) (Park, 1989), Word List Memory (WLM), Word List Recall (WLR), Word List Recognition (WLRc), Constructional Praxis (CP), and Constructional Recall (CR). In addition, total memory (TM) domain scores were obtained by summing scores from the CERAD-K, WLM, WLR, and WLRc. Patients with MCI met Peterson’s criteria of (1) memory complaint corroborated by an informant, (2) objective memory impairment for age, level of education, and sex, (3) essentially preserved general cognitive function, (4) mostly intact functional activities, and (5) no dementia. All MCI patients had an overall Clinical Dementia Rating of 0.5 (Morris, 1993). Classifications of late MCI and early MCI were made as follows. Subjects classified with late MCI reported memory impairment (memory has become worse) and had performance scores greater than 1.5 standard deviations (SDs) below the respective age-specific, education-specific, and sex-specific normative mean on the CERAD-K WLR. On the other hand, subjects classified with early MCI had performance scores between 1.5 and 1.0 SDs below the normative mean. Concise descriptions of the tests and the review process are described in the Supplementary Material. We excluded participants with any history of alcoholism, drug abuse, head trauma, or psychiatric disorders; those taking any psychotropic medications (e.g., cholinesterase inhibitors, antidepressants, benzodiazepines, and antipsychotics); those with multiple vascular risk factors; and those with extensive cerebrovascular disease.

The inclusion criteria of elderly HCs were as follows: (1) subjects were older than 60 years of age; (2) subjects had a performance on the CERAD-K WLR less than 1.0 SDs and on the other domains of the CERAD-K less than 1.5 SDs below the normative mean, and (3) subjects had a Clinical Dementia Rating score of 0. Subjects with any history of alcoholism, drug abuse, head trauma, or psychiatric disorders, as well as those taking any psychotropic medications, were excluded. The study was conducted under the ethical and safety guidelines set forth by the Institutional Review Board of The Catholic University of Korea, which approved all study procedures. Informed and written consent was obtained from all participants.

### MRI Acquisition and Pre-processing

Imaging data were collected in the Department of Radiology of Seoul Saint Mary’s Hospital at The Catholic University of Korea, using a 3T Siemens Verio machine and an eight-channel Siemens head coil (Siemens Medical Solutions, Erlangen, Germany). The parameters used for T1-weighted volumetric magnetization-prepared rapid gradient echo scan sequences were TE = 2.5 ms, TR = 1,900 ms, inversion time = 900 ms, FOV = 250 mm, matrix = 256 × 256, and voxel size = 1.0 mm × 1.0 mm × 1.0 mm.

We processed the data using the VBM8 toolbox^1^, which was part of the SPM8 software package^2^, Wellcome Department of Imaging Neuroscience, London, United Kingdom). Data pre-processing and analysis were performed using SPM8^3^. Data pre-processing involved visual inspection of the T1-weighted images to control for imaging artifacts and the consecutive segmentation into gray matter (GM), white matter (WM), and cerebrospinal fluid (CSF), building a customized template for GM and WM through an iteratively non-linear registration algorithm (DARTEL Toolbox for SPM8) and a normalization of this template to the Montreal Neurological Institute template. The Jacobian determinants resulting from the normalization procedure were used to obtain modulated VBM data preserving regional volumes. Individual GM and WM images were smoothed with an isotropic Gaussian kernel of 6 mm full-width at half-maximum prior to statistical analyses. Global volumes of GM, WM, and CSF were assessed from segmented images using the VBM8 toolbox for SPM8 and summed to generate an estimate for total intracranial volume (TIV).

### Statistical Analysis

Statistical analyses for demographic data were performed with R software (version 2.15.3). Normality assumptions were tested for all continuous variables using the Kolmogorov–Smirnov test. All variables were normally distributed. One-way ANOVA and chi-square (χ^2^) tests were used to assess potential differences between the HC, early, and late MCI groups for all demographic variables. All statistical analyses used a two-tailed level of 0.05 for defining statistical significance. We conducted a whole brain voxel-wise analysis of between-group differences in GM volume with a general linear model (GLM) using SPM8, controlling for age, sex, level of education, and *APOE*ε4 genotype, which are known to affect cortical atrophy (Sowell et al., 2006; Gutiérrez-Galve et al., 2009; Arenaza-Urquijo et al., 2013; Hurtz et al., 2014), and TIV. Multiple corrections were performed using a cluster-extent correction (AlphaSim), as implemented through data processing and analysis for brain imaging (DPABI) (Yan et al., 2016), and the parameters were set as follows: individual voxel threshold *p* < 0.01, number of Monte Carlo simulations = 1000, and *p* < 0.05 as the effective threshold for cluster-extent correction. In *post hoc* procedures, corrected *p* values under a given control procedure for comparing group means of any pairs were calculated (through Studentized range statistics for Turkey-Kramer corrections). The *p* maps were then converted to Z maps according to the normal inverse cumulative distribution function, with the sign of group mean differences applied.

The GM volumes from brain regions with significant group differences were used for further region of interest (ROI) analysis. Resultant raw volumetric measures were normalized to the TIV to compensate for inter-individual variability in head size. To assess the main effect of the interaction between group and cognitive performance on the normalized cortical volume in ROIs, we used multiple regression analyses after adjusting for demographic covariates with a significant difference across the groups. Moreover, we examined the main effect of *APOE* ε4 genotype, cognitive performance, and interaction between two factors on the normalized cortical atrophy within and across the groups.

## Results

### Baseline Demographic and Clinical Data

Table 1 shows the baseline demographic data for the different subject groups. There were no significant differences in sex, number of years of education, and *APOE*ε4 genotype between the control, early, and late MCI groups. However, there was a significant difference in age across groups (*p* < 0.001). As expected based on the inclusion criteria, the groups differed in memory performance test scores (MMSE-K, CERAD-K WLM, WLR, WLRc, and TM) across groups (*p* < 0.001). Concerning non-amnestic cognitive functions, there were also differences in CERAD-K VF, BNT, CR (*p* < 0.001), and CP scores across groups (*p* = 0.024) (see Supplementary Table 1).

**TABLE 1:** Demographic and clinical characteristics of the study participants.

	**Control group (*n* = 37)**	**Early MCI group (*n* = 30)**	**Late MCI group (*n* = 35)**	***P*-value**
Age (years)	73.9 ± 2.0 (72–78)	76.9 ± 4.3 (71–82)	77.3 ± 4.1 (69–82)	<0.001
Sex (M: F,%)	40.5: 59.5	53.3: 46.7	45.7: 54.3	0.579
Education (years)	10.8 ± 4.0 (4–16)	9.0 ± 4.8 (2–20)	9.6 ± 4.3 (2–16)	0.259
*APOE* ε4 carrier (n,%)	8 (21.6%)	13 (43.3%)	14 (40.0%)	0.121
MMSE-K	27.1 ± 1.7 (24–30)	21.7 ± 4.3 (14–30)	21.7 ± 4.5 (11–29)	<0.001
CERAD-K WLM	17.8 ± 3.3 (12–25)	12.8 ± 4.4 (6–20)	10.6 ± 4.1 (3–19)	<0.001
CERAD-K WLR	6.0 ± 1.7 (4–9)	2.5 ± 0.8 (2–5)	0.9 ± 1.0 (0–3)	<0.001
CERAD-K WLRc	9.2 ± 0.8 (8–10)	6.6 ± 2.2 (3–10)	5.2 ± 2.6 (0–10)	<0.001
CERAD-K TM	33.0 ± 5.0 (25–47)	21.9 ± 6.0 (12–32)	16.7 ± 5.7 (6–29)	<0.001

*Data are presented as the mean ± SD (minimum-maximum), unless indicated otherwise. MMSE-K, the Korean version of mini-mental status examination; CERAD-K, the Korean version of the consortium to establish a registry for Alzheimer’s disease; WLM, word list memory; WLR, word list recall; WLRc, word list recognition; TM, total scores of memory domains including the CERAD-K WLM, WLR, WLRc.*

### Group Differences in Normalized Gray Matter Volume

**FIGURE 1:**
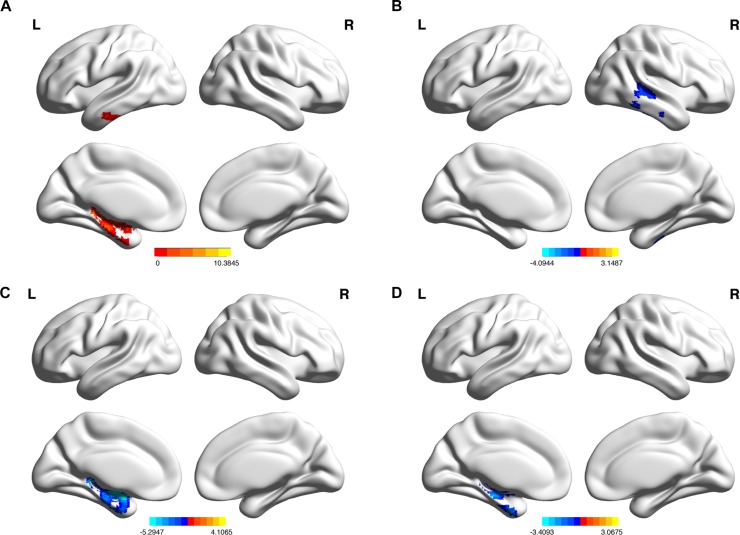
**(A)** Regions of gray matter where normalized volume shows significant differences among the control subjects, early MCI patients, and late MCI patients (Monte Carlo simulation corrected *p* < 0.01). **(B)** Regions of gray matter where normalized volume shows significant reductions in the early MCI group in comparison to the control group. **(C)** In the late MCI group in comparison to the control group. **(D)** In the late MCI group in comparison to the early MCI group (Tukey *post hoc* tests, Monte Carlo simulation corrected *p* < 0.01).

There was a significant group effect on the normalized gray matter volume of the left parahippocampal gyrus and inferior temporal gyrus (Table 2 and Figure 1A, AlphaSim corrected *p* < 0.01). *Post hoc* analysis indicated that the normalized gray matter volumes of the right middle temporal gyrus and left parahippocampal gyrus were significantly lower in early and late MCI groups, respectively, in comparison to the control group (Table 2 and Figures 1B,C), Monte Carlo simulation corrected *p* < 0.01, Tukey *post hoc* tests). In addition, the left fusiform gyrus displayed reduced normalized gray matter volume in the late MCI group in comparison to the early MCI group (Table 2 and Figure 1D), Monte Carlo simulation corrected *p* < 0.01, Tukey *post hoc* tests). Distribution of normalized gray matter volume across groups is demonstrated in Supplementary Figure 1.

**TABLE 2:** Anatomical location of regions showing differences in VBM-based gray matter volume.

**Region**	**L/R**	**Cluster**	**Peak *F* value**	**Peak MNI coordinates (x, y, z)**		
**Group differences in VBM-based gray matter volume among controls, early MCI, and late MCI**						

Parahippocampal gyrus	L	742	7.4368	−18	−36	−3
Inferior temporal gyrus	L	139	6.4856	−66	−24	−27
Tukey *post hoc* tests						
Region	L/R	Cluster	Peak *T* value	Peak MNI coordinates (x, y, z)		
*Early MCI* < *Control*						
Middle temporal gyrus	R	323	−3.0448	51	−27	−15
*Late MCI* < *Control*						
Parahippocampal gyrus	L	2168	−4.5	18	−36	−3
*Late MCI* < *Early MCI*						
Fusiform gyrus	L	871	−3.3645	−24	−3	−39

### A Significant Group by Cognitive Performance Interaction for Normalized Gray Matter Volumes in Regions of Interest

**FIGURE 2:**
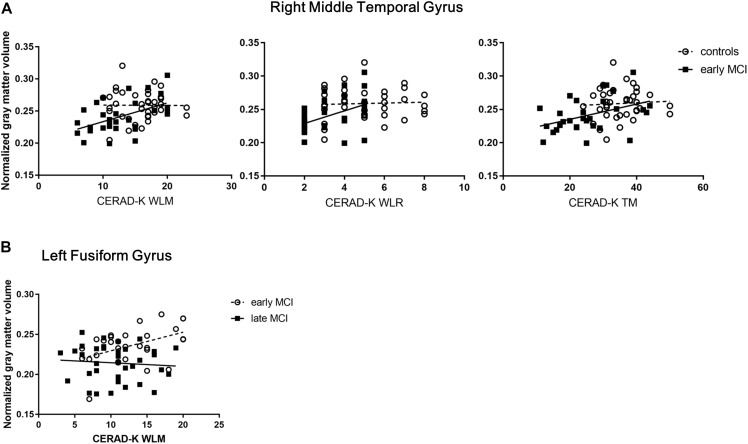
**(A)** Significant group (healthy control vs. early MCI) by memory performance interactions for normalized gray matter volumes of the right middle temporal gyrus (RMTG). **(B)** Significant group (early MCI vs. late MCI) by memory performance interactions for normalized gray matter volumes of the left fusiform gyrus (LFG).CERAD-K, the Korean version of the consortium to establish a registry for Alzheimer’s disease; WLM, word list memory; WLR, word list recall; TM, total scores of memory domains including the CERAD-K WLM, WLR, and WLRc; RMTG, right middle temporal gyrus; LFG, left fusiform gyrus.

After controlling for age covariate, which showed a significant difference across the groups, there was a main effect of the interaction between group (HC vs. early MCI) and memory performance (CERAD-K WLM, WLR, and TM) on the normalized cortical volume of the right middle temporal gyrus (Figure 2A and Supplementary Results 2.1; WLM, Standardized β coefficient (std. β) = 1.14, adjusted *R*^2^ (adj. *R*^2^) = 0.2933, *F* = 7.85, *p* < 0.001; WLR, std. β = 0.55, adj. *R*^2^ = 0.2372, *F* = 7.84, *p* < 0.001; TM, std. β = 1.07, adj. *R*^2^ = 0.2704, *F* = 7.12, *p* < 0.001). There was also a main effect of the interaction between group (early MCI vs. late MCI) and memory performance (CERAD-K WLM) on the normalized gray matter volume of the left fusiform gyrus (Figure 2B and Supplementary Results 2.2; WLM, Standardized β coefficient (std. β) = −0.89, adjusted R^2^ (adj. *R*^2^) = 0.2438, *F* = 7.88, *p* < 0.001). Additionally, there was a trend toward a main effect of interaction between group (early MCI vs. late MCI) and visuospatial function (CERAD-K CP) on the normalized gray matter volume of the left fusiform gyrus (Supplementary Results 2.2; CP, Standardized β coefficient (std. β) = 1.62, adjusted R^2^ (adj. *R*^2^) = 0.1828, *F* = 4.58, *p* = 0.003).

However, there was no main effect of the interaction between group (HC vs. early MCI vs. late MCI) and memory performance on the normalized cortical volume of the left parahippocampal gyrus and inferior temporal gyrus. In addition, there was no main effect of the interaction between group (HC vs. late MCI) and cognitive performance on the normalized gray matter volume of the left parahippocampal gyrus.

### The Main Effect of *APOE* ε4 Genotype, Cognitive Performance, and Interaction Between Two Factors on the Normalized Gray Matter Volume Within and Across the Groups

When analyzed, including all of the controls and the early MCI group, there was a trend toward a main effect of *APOE* ε4 genotype on the normalized gray matter volume of the right middle temporal gyrus. In addition, there was a main effect of the memory performance (CERAD-K WLM) on the normalized cortical volume of the left fusiform gyrus only in case of including all of the controls and the early MCI group. However, there was no main effect of the interaction between *APOE* ε4 genotype and cognitive performance on the normalized gray matter volume in regions of interest (see Supplementary Table 2).

## Discussion

The present study was designed to examine differences in gray matter volume by whole brain voxel-based morphometry and to assess the association between cognitive function evaluated by comprehensive cognitive function test, and cortical thickness in the subdivided trajectory of the prodromal stage in AD.

With respect to the first research question, it was found that the normalized cortical volume of the left parahippocampal gyrus and inferior temporal gyrus showed significant differences across the three groups. In accordance with the present results, previous studies have demonstrated that the left parahippocampal gyrus shows significant atrophy in MCI subjects who later progress to AD (Hämäläinen et al., 2007; Waragai et al., 2009). These results also support previous research indicating more greatly reduced gray matter volume in the left parahippocampal gyrus of AD and late MCI subjects than in healthy elderly subjects and early MCI subjects (Li et al., 2017). Concerning the inferior temporal gyrus, a previous study showed greater atrophy of this region in early and late MCI subjects than in HCs (Li et al., 2017). In addition, another study has reported decreased cortical volumes in the bilateral inferior temporal gyrus of amnestic MCI patients than in healthy elderly subjects (Whitwell et al., 2007).

*Post hoc* analysis of the current study also found that the early MCI group showed greater atrophy in the right middle temporal gyrus in comparison to the HC group. In prior studies, the gray matter volume of this region has been reported to be more greatly decreased in progressive and amnestic MCI patients than in HC subjects (Chetelat et al., 2002; Hämäläinen et al., 2007). Moreover, the cortical volume of this region has been shown to be more greatly reduced in AD patients than in MCI patients (Yao et al., 2011). In another previous, early MCI patients have displayed more temporal lobe atrophy than HCs (Risacher et al., 2013). Additionally, in the current study, greater levels of atrophy in the left fusiform gyrus were found in late MCI subjects in comparison to early MCI subjects. In a previous study, changes in this region have been displayed to predict conversion from MCI to AD (Convit et al., 2000). Furthermore, Aβ retention in hippocampus has been exhibited to be associated with the atrophy of the fusiform gyrus in early stage AD patients (Chang et al., 2016). Finally, it was found that the late MCI group showed reduced gray matter volume in the left parahippocampal gyrus in comparison to the HC group. This result is consistent with that of comparable studies (Hämäläinen et al., 2007; Yao et al., 2011).

The brain regions mentioned above show some overlap with the Braak tau stage III/IV, which is understood to be the primary substrate for amnestic MCI (Markesbery, 2010). These findings may be partly explained by the relationship of tauopathy in Braak-related brain regions with cortical thickness (Wang et al., 2016). Furthermore, these Braak-related regions in stage III/IV have been indicated to predict progression from MCI to AD (Convit et al., 2000). However, in the present study, for brain regions showing more atrophy in early MCI patients than in HCs and for brain regions displaying more greatly reduced cortical volumes in late MCI patients than in early MCI patients and HCs, the observed cortical spreading patterns are mismatched with known cortical spreading patterns in Braak stage III/IV. This intriguing finding might be explained by the fact that the current research was a cross-sectional study and that memory decline does not necessarily represent the progression of the AD pathology, given that early and late MCI are classified based on delayed memory recall scores. Furthermore, the current study did not evaluate tauopathy in any group. In this regard, these findings need to be interpreted with caution, and further longitudinal research evaluating tau pathology needs to be pursued. With respect to atrophy in the middle temporal gyrus, this region of the brain has been shown to constitute part of the default mode network (DMN) (Andrews-Hanna et al., 2010) that has been reported to be associated with Aβ retention and cortical atrophy in the trajectory of AD (Buckner et al., 2005, 2008; Sepulcre et al., 2016).

However, these results differ from some published studies, which have described cortical atrophy in the frontal, parietal cortex, precuneus, hippocampus, and cingulate gyrus (Chetelat et al., 2002; Bozzali et al., 2006; Hämäläinen et al., 2007). This inconsistency in findings may be explained by the fact that previous studies do not classify MCI groups or control for all of the factors affecting atrophy, including age, sex, level of education, and *APOE* ε4 genotype. In addition, it is possible that differences in methodology to examine atrophy levels may account for some observed discrepancies. In other research comparing gray matter volumes in early and late MCI subjects, early MCI patients showed increased volumes in comparison healthy elderly subjects and late MCI patients in the precuneus, supramarginal, and superior parietal gyrus (Li et al., 2017). Given that age affects gray matter volumes (Hurtz et al., 2014), this finding could have been generated by the fact that the mean age of the early MCI group was below 70 years old and significantly younger than the other groups in this previous study.

In addition, the current study showed that the significant interaction between group and memory performance was exhibited in Braak-related brain regions such as the right middle temporal gyrus and left fusiform gyrus. Given the positive relationship between normalized cortical volumes and memory performances in the early MCI group of the present study, a possible explanation for this interaction may be that the gray matter volume of the right middle temporal gyrus decreases more in early MCI subjects than in healthy elderly subjects, and that the cortical volume of the left fusiform gyrus is more greatly reduced in early MCI subjects than in late MCI subjects, when the memory function decreases during progression of AD. Cortical changes in the right middle temporal gyrus, constituting the brain regions of the DMN (Andrews-Hanna et al., 2010), have been reported to be associated with changes in memory (Buckner et al., 2005). Moreover, previous studies evaluating functional changes in this region have suggested that this region might have a compensatory role for AD pathogenesis (Mormino et al., 2011; Wang et al., 2011). In regards to the left fusiform gyrus, the gray matter volume of this brain region has been documented to have a relationship with verbal memory (Bonekamp et al., 2010). In addition, recent study has demonstrated a significant association between the functional connectivity of the fusiform gyrus and MMSE scores in the amnestic MCI patients (Cai et al., 2015a). Furthermore, the existing body of research suggests a compensatory function in the fusiform gyrus by showing increased functional connectivity between the bilateral fusiform gyrus and right thalamus in the early MCI patients rather than in the late MCI patients (Cai et al., 2015b). Although this result might serve as one of the bases for the relatively clear relationship between cortical atrophy of the left fusiform gyrus and cognitive decline in the early MCI group compared with the late MCI group, observed in this study, how the functional brain change contributes to the structural brain change in this brain region is not yet fully understood. Therefore, further studies, which take these variables into account, will need to be undertaken. In addition, there was a trend toward the main effect of interaction between the group and CP on the cortical volume of the left fusiform gyrus in the current study. Previous study has established that AD patients with constructional apraxia have lower volume of the fusiform gyrus than those without constructional apraxia and that AD patients with constructional apraxia show worse performance in other cognitive functions including working memory (Serra et al., 2014).

Moreover, these Braak-related brain regions have been demonstrated to mediate the association between neurodegeneration and cognitive function (Bejanin et al., 2017). Because the current study did not evaluate tau pathology, however, further studies should be undertaken in conjunction with evaluating tau deposition and performing mediation analysis. Moreover, in consideration of previous research reporting that white matter is more sensitive to changes in cognitive function than gray matter (Salat et al., 2010; Meyer et al., 2013), further studies will need to be undertaken to take these variables into account.

Considering the impact of the *APOE* ε4 genotype on the cognition, and cortical atrophy in the MCI (Farlow et al., 2004; Hämäläinen et al., 2008; Risacher et al., 2013), we evaluated the main effect of *APOE* ε4 genotype on the cortical atrophy within and across the groups. In these results, we found a trend toward a main effect of *APOE* ε4 genotype on the cortical volume of the right middle temporal gyrus in case of including both controls and early MCI group, being consistent with those found in the earlier study (Risacher et al., 2013). However, more research including factors such as Aβ and tau protein retention, which affects cortical atrophy, is needed to better understand the possible effect of *APOE* ε4 genotype on the cortical atrophy in the subdivided trajectory of the MCI.

In this investigation, the demographic characteristics of the three groups do not precisely correspond. Notably, the difference in ages across groups is a possible source of error affecting our results. Therefore, these data must be interpreted with caution, and it is necessary to perform additional validation using samples in which the demographic data across groups correspond. Additionally, although the education years were used as a proxy of cognitive reserve in this study, premorbid IQ, a more sensitive marker for cognitive reserve (Schmand et al., 1997), was not evaluated. Moreover, given that this premorbid IQ can affect degree of presentation of memory deficit in the course of the AD (Tuokko et al., 2003), more research using more valid proxy for the cognitive reserve is needed. Finally, with a small sample size, caution must be applied, as the findings might not have sufficient statistical power for evaluating the hypothesis of the present study. Therefore, further research including a larger sample size is needed to be carried out.

In conclusion, this study has explored differences in gray matter volumes and the relationship between cognitive function and cortical thickness across the subdivided prodromal stages of the AD, which show the different prognosis for the AD. We found that Braak-related regions—regions that are vulnerable to tau pathology (Cho et al., 2016; Johnson et al., 2016) — showed significant differences in the normalized cortical volumes across the three groups. Additionally, we found that there was a main effect of the interaction between the subdivided stages of the MCI and memory performance on the cortical volumes in the compensatory regions, which decreased more in early MCI subjects than in HCs and late MCI subjects as memory performance scores declined. The current study has gone some way toward enhancing our understanding of the structural changes in compensatory brain regions to elucidate memory decline in the trajectory of the subdivided prodromal stages of the AD.

## Ethics Statement

This study was conducted under the ethical and safety guidelines set forth by the Institutional Review Board of The Catholic University of Korea, which approved all study procedures. Informed consent was obtained from all participants.

## Author Contributions

DK, HL, and CL conceived and designed the research. DK, HL, SJ, and CL recruited the subjects and followed them to obtain clinical results. DK, NL, and HL performed the *in vivo* MRI experiments. DK and HL performed the image preprocessing and image analysis. NL and SJ performed the statistical analysis. DK wrote the manuscript. CL provided scientific mentorship throughout the project. All authors discussed the results and commented on the manuscript.

## Conflict of Interest Statement

The authors declare that the research was conducted in the absence of any commercial or financial relationships that could be construed as a potential conflict of interest.

## Footnotes

1 http://dbm.neuro.uni-jena.de/vbm8/

2 http://www.fil.ion.ucl.ac.uk/spm/

3 https://www.fil.ion.ucl.ac.uk/spm/software/spm8/
