# Supplementary material for: Differential Associations Between Volumes of Atrophic Cortical Brain Regions and Memory Performances in Early and Late Mild Cognitive Impairment
Source: Front Aging Neurosci. 2019 Sep 4;11:245. doi: 10.3389/fnagi.2019.00245 (PMC6738351; doi:10.3389/fnagi.2019.00245)
Supplement: Supplementary file 1 [file Table_1.DOCX]

Supplementary Material

Differential associations between volumes of atrophic cortical brain regions and memory performances in early and late mild cognitive impairment

**Dong Woo Kang^1^, Hyun Kook Lim^2^, Soo-hyun Joo^1^,** **Na Rae Lee^1^, Chang Uk Lee^1^***

^1^Department of Psychiatry, Seoul St. Mary’s Hospital, College of Medicine, The Catholic University of Korea, Seoul, Republic of Korea

^2^ Department of Psychiatry, Yeouido St. Mary’s Hospital, College of Medicine, The Catholic University of Korea, Seoul, Republic of Korea

*** Correspondence:**Chang Uk Lee, MD, PhD

Department of Psychiatry, Seoul St. Mary’s Hospital, College of Medicine, The Catholic University of Korea, 222, Banpo-daero, Seocho-gu, Seoul, 06591, Republic of Korea

Tel: +82-2-2258-6082, Fax: +82-2-536-8744, E-mail: [jihan@catholic.ac.kr](mailto:jihan@catholic.ac.kr)

# Supplementary Methods

## Neuropsychological evaluation

# Cognitive status was assessed by neuropsychological testing at Seoul St. Mary’s Hospital, The Catholic University of Korea. The cognitive functions of all the subjects were assessed with the Korean version of the Consortium to Establish a Registry for Alzheimer’s Disease (CERAD-K), which included Verbal Fluency (VF), the 15-item Boston Naming Test (BNT), MMSE-K, Word List Memory (WLM), Word List Recall (WLR), Word List Recognition (WLRc), Constructional Praxis (CP), and Constructional Recall (CR). The CERAD is the standardized clinical and neuropsychological assessment battery for the evaluation of patients with Alzheimer's disease. The results were reviewed by a neuropsychologist to determine whether there was evidence of cognitive impairment.

# The VF score is the number of animal names that the subject could name in one minute. The BNT score ranges from 0 to 15 points. The MMSE-K score ranges from 0 to 30 points. The WLM score ranges from 0 to 30 points. The WLR score ranges from 0 to 10 points. The WLR score ranges from 0 to 10 points. The WLRc score ranges from 0 to 10 points. The CP score ranges from 0 to 11 points. The CR score ranges from 0 to 11 points.

## *APOE* genotyping

DNA was isolated from blood using the QIAmp Blood DNA Maxi Kit protocol (Qiagen, Valencia, CA). Genotypes for two APOE SNPs, rs429358 (E*4) and rs7412 (E*2) were determined using TaqMan SNP genotyping assays (Applied Biosystems, Foster City, California).

# Supplementary Results (adjusting for age)

## Significant group by memory performances interactions for normalized gray matter volume of right middle temporal gyrus (controls vs. early MCI)

### A group by CERAD-K WLM interaction was selected as a variable in the finally selected model by stepwise backward regression

| Result of Stepwise Backward Regression  **Analysis of Variance Table** | | | | | | |
| --- | --- | --- | --- | --- | --- | --- |
| Model 1: Normalized volume of the right middle temporal gyrus ~ group + age + CERAD-K WLM + group: CERAD-K WLM Model 2: Normalized volume of the right middle temporal gyrus ~ group + age + CERAD-K WLM + group: CERAD-K WLM | | | | | | |
|  | **Res.Df** | **RSS** | **Df** | **Sum of Sq** | **F** | **Pr(>F)** |
| 1 | 62 | 0.05 |  |  |  |  |
| 2 | 62 | 0.05 | 0 | 0 |  |  |

Finally Selected Model

| **Result of Multiple Regression Analysis** | | | | | | | | | | |
| --- | --- | --- | --- | --- | --- | --- | --- | --- | --- | --- |
|  | **Beta** | **lwr** | **upr** | **SE** | **std.Beta** | **lwr** | **upr** | **SE** | **t value** | ***p*** |
| (Intercept) | 0.40 | 0.29 | 0.51 | 0.06 |  |  |  |  | 7.16 | < 0.001 |
| group | -0.11 | -0.17 | -0.05 | 0.03 | -1.66 | -2.55 | -0.76 | 0.46 | -3.64 | 0.001 |
| age | 0.00 | 0.00 | 0.00 | 0.00 | -0.21 | -0.47 | 0.04 | 0.13 | -1.64 | 0.107 |
| CERAD-K WLM | 0.00 | -0.01 | 0.00 | 0.00 | -0.46 | -0.86 | -0.05 | 0.21 | -2.22 | 0.03 |
| group: CERAD-K WLM | 0.01 | 0.00 | 0.01 | 0.00 | 1.14 | 0.37 | 1.9 | 0.39 | 2.92 | 0.005 |
| *R^2^=0.3362, adj.R^2^=0.2933, F=7.85, p < 0.001, AIC =-282.34* | | | | | | | | | | |

### A group by CERAD-K WLR interaction was selected as a variable in the finally selected model by stepwise backward regression

| Result of Stepwise Backward Regression  **Analysis of Variance Table** | | | | | | |
| --- | --- | --- | --- | --- | --- | --- |
| Model 1: Normalized volume of the right middle temporal gyrus ~ group + CERAD-K WLR + group: CERAD-K WLR Model 2: G2vsG3_Temporal_Mid_R ~ group + age + CERAD-K WLR + group: CERAD-K WLR | | | | | | |
|  | **Res.Df** | **RSS** | **Df** | **Sum of Sq** | **F** | **Pr(>F)** |
| 1 | 63 | 0.05 |  |  |  |  |
| 2 | 62 | 0.05 | 1 | 0 | 1.48 | 0.228 |

Finally Selected Model

| **Result of Multiple Regression Analysis** | | | | | | | | | | |
| --- | --- | --- | --- | --- | --- | --- | --- | --- | --- | --- |
|  | **Beta** | **lwr** | **upr** | **SE** | **std.Beta** | **lwr** | **upr** | **SE** | **t value** | ***p*** |
| (Intercept) | 0.29 | 0.25 | 0.32 | 0.02 |  |  |  |  | 15.82 | < 0.001 |
| group | -0.07 | -0.11 | -0.02 | 0.02 | -0.98 | -1.6 | -0.36 | 0.32 | -3.11 | 0.003 |
| CERAD-K WLR | 0.00 | -0.01 | 0.00 | 0.00 | -0.16 | -0.54 | 0.22 | 0.19 | -0.82 | 0.414 |
| group: CERAD-K WLR | 0.01 | 0.00 | 0.02 | 0.00 | 0.55 | 0.04 | 1.06 | 0.26 | 2.13 | 0.037 |
| *R^2^=0.2718, adj.R^2^=0.2372, F=7.84, p < 0.001, AIC =-278.14* | | | | | | | | | | |

### A group by CERAD-K TM interaction was selected as a variable in the finally selected model by stepwise backward regression

| Result of Stepwise Backward Regression  **Analysis of Variance Table** | | | | | | |
| --- | --- | --- | --- | --- | --- | --- |
| Model 1: Normalized volume of the right middle temporal gyrus ~ group + CERAD-K TM + group: CERAD-K TM Model 2: Normalized volume of the right middle temporal gyrus ~ group + age + CERAD-K TM + group: CERAD-K TM | | | | | | |
|  | **Res.Df** | **RSS** | **Df** | **Sum of Sq** | **F** | **Pr(>F)** |
| 1 | 62 | 0.05 |  |  |  |  |
| 2 | 62 | 0.05 | 0 | 0 |  |  |

Finally Selected Model

| **Result of Multiple Regression Analysis** | | | | | | | | | | |
| --- | --- | --- | --- | --- | --- | --- | --- | --- | --- | --- |
|  | **Beta** | **lwr** | **upr** | **SE** | **std.Beta** | **lwr** | **upr** | **SE** | **t value** | ***p*** |
| (Intercept) | 0.41 | 0.28 | 0.53 | 0.06 |  |  |  |  | 6.45 | < 0.001 |
| group | -0.11 | -0.18 | -0.04 | 0.03 | -1.63 | -2.65 | -0.62 | 0.52 | -3.15 | 0.002 |
| age | 0.00 | 0.00 | 0.00 | 0.00 | -0.22 | -0.49 | 0.04 | 0.14 | -1.64 | 0.106 |
| CERAD-K TM | 0.00 | 0.00 | 0.00 | 0.00 | -0.48 | -0.95 | 0 | 0.24 | -1.96 | 0.055 |
| group: CERAD-K TM | 0.00 | 0.00 | 0.01 | 0.00 | 1.07 | 0.24 | 1.9 | 0.42 | 2.51 | 0.015 |
| *R^2^=0.3146, adj.R^2^=0.2704, F=7.12, p < 0.001, AIC =-280.2* | | | | | | | | | | |

## A significant group by memory performances interactions for normalized gray matter volume of left fusiform gyrus (earl MCI vs. late MCI)

### A group by CERAD-K WLM interaction was selected as a variable in the finally selected model by stepwise backward regression

| Result of Stepwise Backward Regression  **Analysis of Variance Table** | | | | | | |
| --- | --- | --- | --- | --- | --- | --- |
| Model 1: Normalized volume of the left fusiform gyrus ~ group + CERAD-K WLM + group: CERAD-K WLM Model 2: Normalized volume of the left fusiform gyrus ~ group + age + CERAD-K WLM + group: CERAD-K WLM | | | | | | |
|  | **Res.Df** | **RSS** | **Df** | **Sum of Sq** | **F** | **Pr(>F)** |
| 1 | 61 | 0.03 |  |  |  |  |
| 2 | 60 | 0.03 | 1 | 0 | 0.79 | 0.3779 |

Finally Selected Model

| **Result of Multiple Regression Analysis** | | | | | | | | | | |
| --- | --- | --- | --- | --- | --- | --- | --- | --- | --- | --- |
|  | **Beta** | **lwr** | **upr** | **SE** | **std.Beta** | **lwr** | **upr** | **SE** | **t value** | ***p*** |
| (Intercept) | 0.19 | 0.14 | 0.25 | 0.03 |  |  |  |  | 7.38 | < 0.001 |
| group | 0.01 | -0.02 | 0.04 | 0.02 | 0.27 | -0.37 | 0.9 | 0.32 | 0.82 | 0.415 |
| CERAD-K WLM | 0.01 | 0.00 | 0.01 | 0.00 | 0.9 | 0.21 | 1.6 | 0.35 | 2.56 | 0.013 |
| group: CERAD-K WLM | 0.00 | -0.01 | 0.00 | 0.00 | -0.89 | -1.69 | -0.1 | 0.41 | -2.19 | 0.032 |
| *R^2^=0.2793, adj.R^2^=0.2438, F=7.88, p < 0.001, AIC =-309.81* | | | | | | | | | | |

Res. Df, residual degrees of freedom; RSS, residual sum of squares; Df, degree of freedom; Sq, squares; Pr (> F), Probability of F value. WLM, Word List Memory; WLR, Word List Recall; TM, total scores of memory domains including CERAD-K WLM, WLR, WLRc.

### A group by CERAD-K CP interaction was selected as a variable in the finally selected model by stepwise backward regression

| Result of Stepwise Backward Regression  **Analysis of Variance Table** | | | | | | |
| --- | --- | --- | --- | --- | --- | --- |
| Model 1: Normalized volume of the left fusiform gyrus ~ group + age + CERAD-K CP + group: CERAD-K CP Model 2: Normalized volume of the left fusiform gyrus ~ group + age + CERAD-K CP + group: CERAD-K CP | | | | | | |
|  | **Res.Df** | **RSS** | **Df** | **Sum of Sq** | **F** | **Pr(>F)** |
| 1 | 60 | 0.1 |  |  |  |  |
| 2 | 60 | 0.1 | 1 | 0 | 0 |  |

Finally Selected Model

| **Result of Multiple Regression Analysis** | | | | | | | | | | |
| --- | --- | --- | --- | --- | --- | --- | --- | --- | --- | --- |
|  | **Beta** | **lwr** | **upr** | **SE** | **std.Beta** | **lwr** | **upr** | **SE** | **t value** | ***p*** |
| (Intercept) | 0.64 | 0.41 | 0.87 | 0.12 |  |  |  |  | 5.53 | < 0.001 |
| group | -0.16 | -0.29 | -0.03 | 0.72 | -1.75 | -3.18 | -0.31 | 0.73 | -2.38 | 0.02 |
| age | 0.00 | 0.00 | 0.00 | 0.00 | -0.17 | -0.39 | 0.06 | 0.11 | -1.46 | 0.15 |
| CERAD-K CP | -0.02 | -0.04 | 0.00 | 0.01 | -0.69 | -1.38 | -0.01 | 0.35 | -1.99 | 0.051 |
| group: CERAD-K CP | 0.01 | 0.00 | 0.03 | 0.01 | 1.62 | -0.05 | 3.29 | 0.85 | 1.91 | 0.061 |
| *R^2^=0.2338, adj.R^2^=0.1828, F=4.58, p =0.003, AIC =-225.01* | | | | | | | | | | |

Res. Df, residual degrees of freedom; RSS, residual sum of squares; Df, degree of freedom; Sq, squares; Pr (> F), Probability of F value. WLM, Word List Memory; WLR, Word List Recall; CP, Constructional Praxis; TM, total scores of memory domains including CERAD-K WLM, WLR, WLRc.

# Supplementary Figures and Tables

## Supplementary Figures


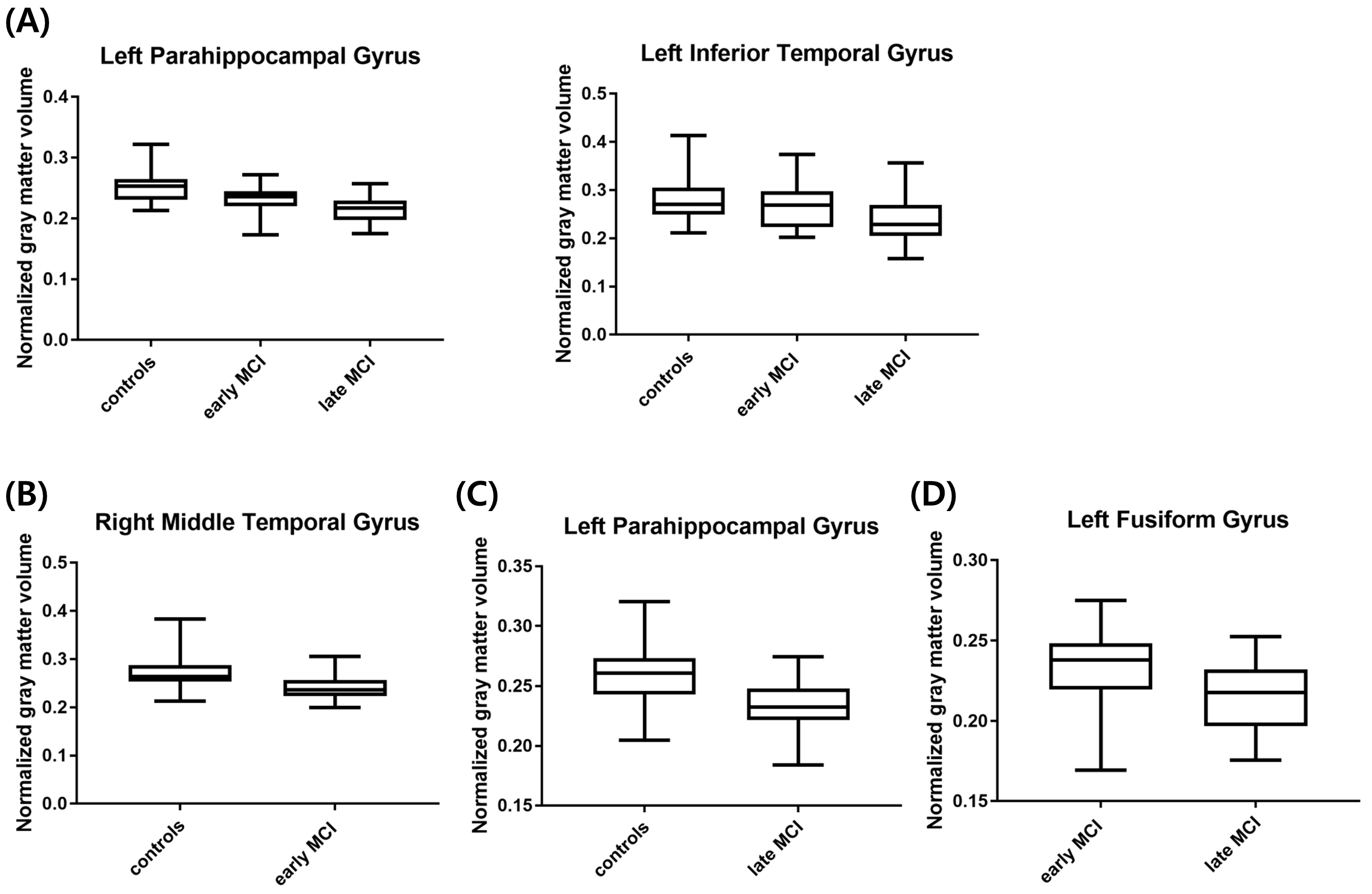


**Supplementary Figure 1.** (A) The differences in normalized gray matter volumes among the control, early, and late MCI groups. Post-hoc analysis, (B) between the control and early MCI groups, (C) between the control and late MCI groups, (D) between the early and late MCI groups.

## Supplementary Tables

|  | Control group (n=37) | Early MCI group (n=30) | Late MCI group (n=35) | *P* value |
| --- | --- | --- | --- | --- |
| CERAD-K VF | 15.0 ± 3.5 (10-23) | 10.4 ± 3.7 (6-20) | 10.2 ± 3.1 (6-17) | < 0.001 |
| CERAD-K BNT | 12.6 ± 1.7 (9-15) | 9.2 ± 3.0 (4-15) | 8.7 ± 2.8 (2-14) | < 0.001 |
| CERAD-K CP | 10.5 ± 0.7 (9-11) | 9.2 ± 1.7 (5-11) | 9.7 ± 1.3 (7-11) | 0.024 |
| CERAD-K CR | 7.2 ± 2.3 (3-11) | 2.8 ± 3.0 (0-10) | 2.1 ± 1.9 (0-6) | < 0.001 |

**Supplementary Table 1.** Non-amnestic cognitive function of the study participants. Data are presented as the means ± SD (minimum-maximum) unless indicated otherwise. CERAD-K, Korean version of Consortium to Establish a Registry for Alzheimer’s Disease; VF, Verbal Fluency; BNT, 15-item Boston Naming Test; CP, Constructional praxis; CR, Constructional Recall.

| Normalized gray matter volume of the regions of interest  (Related cognitive domain ) | Group | Adj. R^2^ | F value |  | *P* value of explanatory variables | | |
| --- | --- | --- | --- | --- | --- | --- | --- |
|  |  |  |  |  | *APOE* ε4 | Cognitive performance | *APOE* ε4 x Cognitive performance |
| Right Middle Temporal Gyrus  (CERAD-K WLM) | Controls | 0.0216 | 0.81 |  | 0.863 | 0.1 | 0.862 |
|  | Early MCI | 0.3024 | 4.14 |  | 0.933 | 0.101 | 0.597 |
|  | Controls and early MCI | 0.1359 | 3.6 |  | 0.186† | 0.798 | 0.356 |
| Right Middle Temporal Gyrus  (CERAD-K WLR) | Controls | 0.0896 | 0.26 |  | 0.916 | 0.355 | 0.889 |
|  | Early MCI | 0.2381 | 3.27 |  | 0.61 | 0.415 | 0.658 |
|  | Controls and early MCI | 0.1467 | 3.84 |  | 0.083† | 0.724 | 0.224 |
| Right Middle Temporal Gyrus  (CERAD-K TM) | Controls | 0.0473 | 0.59 |  | 0.972 | 0.164 | 0.975 |
|  | Early MCI | 0.2801 | 3.82 |  | 0.94 | 0.15 | 0.474 |
|  | Controls and early MCI | 0.1426 | 3.74 |  | 0.24† | 0.482 | 0.428 |
| Left Fusiform Gyrus  (CERAD-K WLM) | Early MCI | 0.2557 | 3.49 |  | 0.903 | 0.131 | 0.628 |
|  | Late MCI | 0.0636 | 0.49 |  | 0.204 | 0.382 | 0.222 |
|  | Early and late MCI | 0.0343 | 1.57 |  | 0.55 | 0.41* | 0.764 |

**Supplementary Table 2.** Effect of *APOE* ε4 genotype, cognitive performance, and interaction between two factors on the normalized gray matter volumes in the regions of interest. Multiple regression analysis adjusted with age. *, *p* < 0.05 in the finally selected model by backward stepwise regression analysis. †, 0.05 < *p* < 0.1 in the finally selected model by backward stepwise regression analysis (a trend toward a main effect of explanatory variables). CERAD-K, Korean version of Consortium to Establish a Registry for Alzheimer’s Disease; WLM, Word List Memory; WLR, Word List Recall; TM, total scores of memory domains including of CERAD-K WLM, WLR, WLRc.
